# Long Chain Fatty Acid Acylated Derivatives of Quercetin-3-*O*-Glucoside as Antioxidants to Prevent Lipid Oxidation

**DOI:** 10.3390/biom4040980

**Published:** 2014-11-06

**Authors:** Sumudu N. Warnakulasuriya, Ziaullah, H.P. Vasantha Rupasinghe

**Affiliations:** Department of Environmental Sciences, Faculty of Agriculture, Dalhousie University, Truro, NS B2N 5E3, Canada; E-Mails: sumudunw@dal.ca (S.N.W.); Ziaullah.ziaullah@dal.ca (Z.)

**Keywords:** Q3G esters, fatty acids, antioxidant, lipid oxidation, LDL oxidation

## Abstract

Flavonoids have shown promise as natural plant-based antioxidants for protecting lipids from oxidation. It was hypothesized that their applications in lipophilic food systems can be further enhanced by esterification of flavonoids with fatty acids. Quercetin-3-*O*-glucoside (Q3G) was esterified individually with six selected long chain fatty acids: stearic acid (STA), oleic acid (OLA), linoleic acid (LNA), α-linolenic acid (ALA), eicosapentaenoic acid (EPA) and decosahexaenoic acid (DHA), using *Candida antarctica* B lipase as the biocatalyst. The antioxidant activity of esterified flavonoids was evaluated using lipid oxidation model systems of poly-unsaturated fatty acids-rich fish oil and human low density lipoprotein (LDL), *in vitro*. In the oil-in-water emulsion, Q3G esters exhibited 50% to 100% inhibition in primary oxidation and 30% to 75% inhibition in secondary oxidation. In bulk oil, Q3G esters did not provide considerable protection from lipid oxidation; however, Q3G demonstrated more than 50% inhibition in primary oxidation. EPA, DHA and ALA esters of Q3G showed significantly higher inhibition in Cu^2+^- and peroxyl radical-induced LDL oxidation in comparison to Q3G.

## 1. Introduction

There has been a strong interest in nutritional benefits of omega-3 (n-3) poly-unsaturated fatty acids (PUFA) containing dietary lipids due to their clinical benefits associated with prevention of chronic diseases, such as cardiovascular diseases and cancer [1,2]. However, foods rich in PUFA are highly susceptible to oxidation, leading to rancidity and nutritional loss [2,3]. The oxidation of dietary unsaturated fatty acids results in generation of advanced lipid oxidation end-products which are in part cytotoxic and genotoxic [4].

Lipid oxidation can occur at the cellular level, in the plasma membrane, as well as in low density lipoprotein (LDL)-cholesterols, due to the presence of cellular reactive oxygen species and is responsible for many pathological conditions [5]. The oxidation of PUFA within the forms of oxidized LDL or atherogenic LDL and therefore, is a causal factor for pathogenesis of atherosclerosis [6]. Habitual consumption of dietary antioxidants is one of the ways to retard the oxidative stress mediated chronic diseases [7]. Potential safety risks associated with the synthetic antioxidants has created an increasing market demand for natural antioxidants [3]. Many natural polyphenols including flavonoids are abundant mainly in plant foods, such as fruits and their products. However, lipophilicity of an antioxidant molecule is important for its effectiveness when incorporated into different food matrices [6].

The moderately hydrophilic nature of naturally occurring flavonoid glycosides, could become a limiting factor when incorporating them into more lipophilic food systems [8,9]. The structural modification of flavonoid molecules is one of the possible approaches to enhance their incorporation in lipophilic food products such as fish oil. The preparation of lipophilic derivatives of flavonoids using aliphatic molecules has shown greater miscibility in lipophilic systems [10,11]. Enzymatic acylation of natural polyphenols with long chain fatty acids has been suggested for improving the lipophilic nature of the glycosylated flavonoids [8,12,13,14]. The present study is aimed at evaluating the ability of six unsaturated, mono- and poly-unsaturated long chain fatty acid derivatives of Q3G in inhibiting oxidation of PUFA in lipid food model systems of oil-in-water emulsions and bulk oil and inhibition of Cu^2+^- and peroxyl radical-induced oxidation of human LDL *in vitro*.

## 2. Material and Methods

### 2.1. Chemicals and Supplies

Ferric chloride, dimethyl sulfoxide, 2,2'-azobis(2-amidinopropane) dihydrochloride (AAPH), trichloroacetic acid (TCA), 2-thiobarbituric acid (TBA), butylated hydroxytoluene (BHT), molecular sieves and *Candida antactica* B lipase were obtained from Sigma-Aldrich (Oakville, ON, Canada). Acetic acid, hydrochloric acid, HPLC grade chloroform, and HPLC grade methanol were purchased from Fisher Scientific (Ottawa, ON, Canada). Ethanol anhydrous was obtained from Commercial Alcohols (Montreal, QC, Canada). Round bottom 96-well plates were purchased from Corning Incorporated (Edison, NY, USA). Quercetion-3-*O*-glucoside (Q3G) was obtained from Indofine Chemical Company (Hillsborough, NJ, USA). Bulk fish oil was a generous gift from BASF A/S (Malmparken 5, Ballerup, Denmark). The composition of the oil was 19.1% *cis*-monounsaturates and 33.7% *cis*-polyunsaturates (29.9% ω-3 polyunsaturated fatty acids, 3.7% ω-6 polyunsaturated fatty acids), 1.2% *trans* fatty acids, 25.6% saturated fatty acids, 5.6% EPA, 22.9% DHA by weight). LDL isolated from human plasma (in 150 mM NaCl, 0.01% EDTA, pH 7.4) was purchased from EMD Chemicals Inc. (Gibbstown, NJ, USA). Free fatty acids were purchased from Nu-Check-prep, Inc. (Waterville, MN, USA). All other chemicals were purchased from Fisher Scientific.

### 2.2. Synthesis of Fatty Acid Acylated Derivatives of Q3G

**Figure 1:**
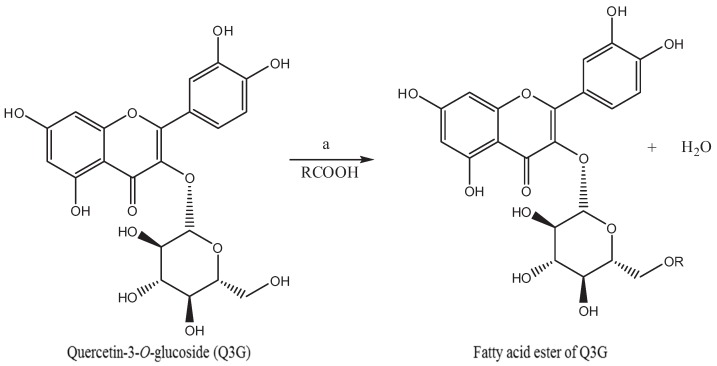
Esterification of Q3G with acyl donor fatty acids. (**a**) Acetone, 3 °A molecular sieves, *Candida antactica* B lipase, 45 °C, Stirring, 24 h; R = Oleic acid, Stearic acid, Linoleic acid, α-Linolenic, Eicosapentaenoic acid, and Docosahexaenoic acid.

Synthesis of fatty acid esters of Q3G (phenolipids) was carried out through enzymatic esterification of Q3G separately with stearic acid (STA), oleic acid (OLA), linoleic acid (LNA), α-linolenic acid (ALA), eicosapentaenoic acid (EPA) and decosahexaenoic acid (DHA) as acyl donors as previously described [14] (Figure 1). Briefly, Q3G (500 mg) and each acyl donor were added into a reaction vessel containing dried 3 Å molecular sieves in a molar ratio of flavonoid:acyl donor 1:5. Anhydrous acetone was used as the solvent. The acylation was initiated by adding *Candida antarctica* B immobilised lipase (2 g) as the biocatalyst. Then, the mixture was incubated at 45 °C while stirring for approximately 48 h in a sand bath. Enzymatic conversion of the substrate was qualitatively monitored periodically by TLC analysis using silica gel plates (TLC Silica gel 60F_254_–Aluminum sheets 20 cm × 20 cm, Merck KGaA, Darmstadt, Germany). Acetone:toluene (50:50 v:v) solvent mixture was used as the TLC solvent system, with the addition of few drops of glacial acetic acid and visualized under UV light and iodide staining. After confirming the completion, the enzymatic reaction was halted by filtering the immobilized lipase and molecular sieves from the reaction mixture and the acetone was removed by vacuum evaporation. The synthesized phenolipids were isolated by subjecting the crude product to silica gel column chromatography using acetone:toluene; 40:60 to 50:50. Preparative TLC was performed under the same conditions as above.

### 2.3. Determination of Primary Oxidation in Bulk Fish Oil Model System

Different concentrations of Q3G and fatty acid acylated derivatives of Q3G (0.5, 1, 5, and 10 mmol·L^−1^), dissolved in 1% dimethyl sulfoxide, were mixed with bulk fish oil and incubated at 40 °C for 3 and 5 days. The lipid peroxides which were formed during lipid oxidation were determined as the peroxide value using acetic acid-chloroform method [15]. Briefly, oxidized fish oil was dissolved in 3:2 ratio of acetic acid–chloroform mixture and 0.5 mL of freshly prepared saturated KI solution was added and gently mixed. After 1 min, 30 mL of deionized water was added, followed by addition of 1 mL of starch. The liberated iodine was titrated with 0.1 N of Na_2_S_2_O_3_. Percentage inhibition of lipid peroxidation was calculated, based on the peroxide values obtained.

### 2.4. Preparation of Aqueous Emulsion Model System

An aqueous emulsion (oil-in-water) of fish oil was prepared following a method described previously [16]. An emulsion of 10 mg of fish oil per 1 mL of buffer (pH 7) containing 0.05 M tris HCl, 0.15 M KCl and 4% Tween 20 as an emulsifier, was prepared by homogenizing the mixture using a homogenizer (model PCU, Polytron®, Luzernerstrasse, Littau-Luzern, Switzerland), at 4.5 speed for 30 s.

### 2.5. Determination of Primary Oxidation in Aqueous Emulsion Model System

The procedure for ferric thiocyanate test was followed [16]. Ethanolic solutions (95% ethanol) of Q3G and its esters in 0.5, 1, 5, and 10 mmol·L^−1^ concentrations were prepared in disposable 13 × 100 mm borosilicate glass tubes and the solvent was completely evaporated under nitrogen flow. After solubilising the dried compounds with 10 µL ethanol, 80 µL of emulsion was added and vortexed. Oxidation was induced by adding 10 µL of peroxyl radical generator, AAPH and incubated at room temperature for 40 min. At the end of the incubation, further oxidation was halted immediately by adding 10 µL of 1000 mg·L^−1^ BHT into all the samples. Samples (30 µL) were diluted with 210 µL of 75% ethanol and 30 µL of 3% NH_4_SCN was added. After 3 min, 30 µL of 2 mmol·L^−1^ ferric chloride in 3.5% HCl was added and absorbance was measured at 495 nm in 96-well microplates, using FLUOstar OPTIMA microplate reader (BMG Labtech, Durham, NC, USA). Blank samples were prepared for all the concentration levels of all the compounds, with no addition of emulsion, in order to eliminate the absorbance contributed by compounds themselves and blank absorbance values were subtracted from the sample absorbance. Percentage inhibition of lipid oxidation was calculated. The hydroperoxides, formed during lipid oxidation, react with ferrous ions to produce ferric ions, which are detected as the ferric thiocyanate red chromogen.

### 2.6. Determining the Inhibition of Secondary Oxidation

Secondary lipid oxidation, in both bulk fish oil and aqueous emulsion (oil-in-water) model systems, were determined by thiobarbituric acid reactive substances (TBARS) assay [16,17]. Desired concentrations of Q3G and fatty acid derivatives of Q3G in 95% ethanol (0.5, 1, 5, and 10 mmol·L^−1^) were placed in disposable 13 × 100 mm borosilicate glass tubes and the solvent was completely evaporated under nitrogen flow. Twenty microliters of ethanol and 80 µL of bulk fish oil or aqueous emulsion were added and vortexed well to ensure complete dispersion of the compounds in the bulk fish oil or aqueous emulsions. Induction of oxidation was achieved by heating the glass tubes, covered with breathable caps, for 3 h at 50 °C in a shaking incubator (model Apollo HP50, CLP Tools, San Diego, CA, USA).

The TBA reagent (0.375% TBA and 15% TCA in 0.25 M HCl) was added to all the oxidized fish oil or emulsion samples as well as the controls. After vortexing, all the samples were capped and placed in a water bath (Isotemp 205, Fisher scientific, Ubuque, IA, USA) at 80 °C for 15 min. The sample tubes were allowed to cool to room temperature and 2 mL of 1-butanol was added to each sample, followed by vortexing and centrifuging (IEC International Centrifuge, MA, USA) at 40× g for 20 min. After loading the supernatant (1-butanol layer) into 96-well microplates, fluorescence intensity (FI) was measured for excitation at 532 nm and emission at 580, using FLUOstar OPTIMA plate reader (BMG Labtech). The percentage inhibition of the samples was calculated in comparison to controls with no antioxidants, using the equation below:

% Inhibition = 100 (FI _control_ − FI _sample_)/FI _control_

### 2.7. LDL Oxidation

#### 2.7.1. LDL Preparation

The assay was conducted as described previously [18]. Briefly, prior to dialysis of LDL, cellulose dialysis tubing (Thermo Fisher Scientific Inc., Ottawa, ON, Canada) was pretreated to remove inherent antioxidants. The tubing was cut into the desired length (6–8 cm), soaked in deionized water for 15 min and heated at 80 °C in a 10 mmol·L^−1^ sodium bicarbonate solution for 30 min while stirring. It was transferred into a 10 mmol·L^−1^ Na_2_EDTA solution and soaked for 30 min. Again, the tubing was transferred to the deionized water at 80 °C and soaked for 30 min while stirring. The membrane was cooled and submerged in 50% ethanol solution and refrigerated. To perform the dialysis, the cellulose tubing was washed both inside and outside first with deionized water and then with 10 mmol·L^−1^ PBS solution containing 0.138 M NaCl and 0.0027 M KCl (pH 7.4, at 25 °C). One end of the tubing was sealed, LDL was then injected into the open end which was then sealed completely. The sealed tubing was then immersed in 10 mmol·L^−1^ PBS solution at 4 °C for 24 h. Buffer was changed every six hours. Once the dialysis was completed, the dialyzed LDL was immediately transferred into an amber colored vial and stored at −80 °C for use within two weeks.

#### 2.7.2. Determination of Protein Content of Dialysed LDL

Protein content of the dialyzed LDL was determined using the Lowry’s method [19]. Bovine serum albumin was used as the standard protein source. Lowry’s reagent (1 mL) was added to the blank, standards and dialyzed LDL and left for 20 min. Then, Folin Ciocalteu reagent (0.5 mL) was added, followed by immediate thorough mixing and allowed to stand for 30 min. Absorbance was measured at 750 nm. Protein content of the LDL sample was calculated using the calibration curve of BSA (10, 20, 40 and 50 µg/mL). The LDL sample was diluted appropriately with PBS to reach 100 µg/mL protein content.

#### 2.7.3. Inhibition of LDL Oxidation

Oxidation was induced by two methods: using copper(II) sulfate and peroxyl radical generator, AAPH. Different concentrations (1, 10, 100, and 500 µmol·L^−1^) of Q3G derivatives and Q3G were prepared in methanol. Dialyzed LDL (160 µL) was mixed with 20 µL of the test compound in borosilicate glass tubes and 20 µL of Cu^2+^ (10 µM )/AAPH (5 mM) was added. All the samples were incubated at 37 °C for 4 h in a shaking water bath. Solutions of 1 mmol·L^−1^ EDTA and BHT were added to terminate oxidation in Cu^2+^ and peroxy radical-induced systems, respectively. LDL oxidation was determined using the TBARS assay [20].

### 2.8. Statistical Analysis

The statistical analysis was carried out using SAS 9.2 and Minitab 16 statistical software packages. The effect of the two factors: “type of test compound” and “concentration”, on percentage inhibition of lipid oxidation was determined using a two factor factorial design of three replicates. The factor, “type of test compound”, contained six levels (six different test compounds) and the factor “concentration” contained four levels (four different concentrations). The data was analyzed by ANOVA using general linear model. Multiple means comparison was carried out by least square means to identify the significant differences across the test compounds for each concentration. When there are no significant interactions between the two factors; “type of test compound” and “concentration”, the significant differences among the effects of “type of test compound” were determined regardless of the concentration, using Tukey’s multiple means comparison method. A probability of *p* ≤ 0.05 was taken as statistically significant.

## 3. Results

### 3.1. Inhibition of Oxidation in Bulk Fish Oil and Aqueous Emulsion

In n-3 PUFA-rich bulk fish oil, the parent compound Q3G showed the highest percentage inhibition of primary oxidation over all the fatty acid derivatives of Q3G at 10 mmol·L^−1^. However, the ALA, EPA and DHA derivatives of Q3G demonstrated considerable inhibition of 30% to 40% (Table 1). When the secondary oxidation products in the bulk fish oil system was measured, all the tested compounds, except for the EPA and DHA derivatives of Q3G, demonstrated a concentration dependent effect where the higher concentrations provided the higher percent inhibition. Q3G esterified to LNA exhibited the highest percentage inhibition of 70% at the concentration of 10 mmol·L^−1^ (Table 2). In general, the synthesized phenolipids had no significant (*p* ≤ 0.05) effect, compared to their parent flavonoid Q3G, on inhibition of both primary and secondary oxidation in bulk fish oil model system.

In primary lipid oxidation in oil-in-water emulsion (Table 3), all the six esters of Q3G exhibited better antioxidant properties than the parent compound Q3G, showing more than 50% inhibition in two or more tested concentration levels in the range of 0.5–10 mmol·L^−1^. The STA, OLA and LNA derivatives of Q3G were effective, with more than 50% inhibition in all the tested concentrations, showing that they are useful as antioxidants, even at low concentration levels such as 0.5 mmol·L^−1^. More than 40% inhibition was observed in the EPA and DHA derivatives in all the tested concentrations and DHA derivative achieved 100% inhibition at 10 mmol·L^−1^. In secondary oxidation of oil-in-water emulsion, all the phenolipids had their highest inhibition at 10 mmol·L^−1^ and there was more than 30% inhibition (Table 3). Overall, the results showed that in the oil-in-water emulsion, Q3G stearate showed the greatest antioxidant protection since it reached the greatest inhibition at 5 mmol·L^−1^ in the primary oxidation. In the case of secondary oxidation, Q3G stearate is the best antioxidant at 10 mmol·L^−1^. The parent compound Q3G showed lower inhibition than the phenolipids, even at its most effective concentration level of 10 mmol·L^−1^. It can be inferred that in the oil-in-water emulsion system, the phenolipids prepared by esterification of the fatty acids and Q3G have a better antioxidant activity than Q3G.

**Table 1:** Inhibition of primary lipid oxidation in bulk fish oil in relation to concentration of the test compounds.

Compound	% Inhibition (Relative to the Control)							
	Day 3				Day 5			
	Concentration (mmol·L^−1^)				Concentration (mmol·L^−1^)			
	0.5	1	5	10	0.5	1	5	10
Q3G	0 ± 0.0**c**	0 ± 0.0**c**	45 ± 7.1**bc**	57 ± 8.7**a**	8 ± 1.5**D**	12 ± 1.4**CD**	34 ± 4.4**BC**	52 ± 6.3**A**
Q3G stearate	0 ± 0.0**c**	0 ± 0.0**c**	24 ± 2.0**bc**	26 ± 4.0**bc**	13 ± 6.0**CD**	22 ± 2.6**CD**	34 ± 2.3**BC**	29 ± 3.2**BC**
Q3G oleate	7 ± 2.8**c**	0 ± 0.0**c**	14 ± 6.5**c**	12 ± 9.3**c**	0 ± 0.0**D**	6 ± 3.8**CD**	26 ± 6.7**CD**	30 ± 15.2**BC**
Q3G linoleate	18 ± 8.4**bc**	16 ± 13.4**bc**	14 ± 0.2**c**	21 ± 2.7**bc**	11 ± 1.0**CD**	22 ± 3.4**CD**	28 ± 3.0**C**	26 ± 5.6**C**
Q3G α-linolenate	14 ± 8.2**c**	5 ± 3.5**c**	12 ± 6.4**c**	17 ± 3.4**bc**	5 ± 3.5**D**	11 ± 4.8**CD**	22 ± 4.1**CD**	40 ± 2.7**AB**
Q3G eicosapentaenoate	18 ± 7.5**bc**	21 ± 4.7**bc**	34 ± 3.8**b**	30 ± 5.0**bc**	16 ± 2.6**CD**	18 ± 0.5**CD**	37 ± 2.1**B**	34 ± 1.6**BC**
Q3G docosahexaenoate	9 ± 7.0**c**	17 ± 1.0**bc**	26 ± 0.8**bc**	21 ± 1.2**bc**	19 ± 2.1**CD**	21 ± 6.2**CD**	30 ± 8.8**BC**	38 ± 1.3**AB**

Different concentrations of Q3G and fatty acid acylated derivatives of Q3G were incorporated into bulk fish oil and incubated at 40 °C for 3 and 5 days. The lipid peroxides formed were determined using acetic acid-chloroform method and % inhibition of lipid peroxidation was presented as mean ± standard deviation. Data from day 3 and day 5 were statistically analyzed separately. Different letters (a,b,c… and A,B,C…) denote significant differences among test compounds and concentrations, for day 3 and day 5, respectively (*p* ≤ 0.05).

**Table 2:** Inhibition of secondary lipid oxidation in bulk fish oil in relation to the concentration of the test compounds.

Compound	% Inhibition (Relative to the Control)			
	Concentration (mmol·L^−1^)			
	**0.5**	**1**	**5**	**10**
Q3G	24 ± 5.8**e**	35 ± 2.4**d**	60 ± 2.5**b**	69 ± 0.1**a**
Q3G stearate	39 ± 4.4**d**	35 ± 1.2**d**	46 ± 1.2**cd**	52 ± 2.1**bc**
Q3G oleate	40 ± 1.4**cd**	60 ± 0.7**b**	64 ± 1.5**ab**	67 ± 1.7**ab**
Q3G linoleate	34 ± 3.0**d**	49 ± 3.6**c**	63 ± 1.6**ab**	70 ± 0.6**a**
Q3G α-linolenate	38 ± 1.0**d**	33 ± 4.4**de**	42 ± 0.6**cd**	51 ± 0.9**bc**
Q3G eicosapentaenoate	46 ± 2.6**cd**	40 ± 1.9**cd**	50 ± 4.1**bc**	31 ± 1.1**de**
Q3G docosahexaenoate	50 ± 4.2**cd**	50 ± 3.5**bc**	47 ± 6.4**cd**	33 ± 2.5**de**

Induction of secondary oxidation in bulk fish oil was achieved by incubating for 3 h at 50 °C in a shaking incubator and oxidation was determined by TBARS assay. Data were presented as mean ± standard deviation. Different letters (a,b,c…) denote significant differences among test compounds for all concentrations (*p* ≤ 0.05).

**Table 3:** Inhibition of lipid oxidation in oil-in-water emulsion in relation to the concentration of the test compounds.

Compound	% Inhibition (Relative to the Control)							
	Primary Oxidation				Secondary Oxidation			
	Concentration (mmol·L^−1^)				Concentration (mmol·L^−1^)			
	0.5	1	5	10	0.5	1	5	10
Q3G	29 ± 6.7**c**	39 ± 9.4**bc**	42 ± 9.1**bc**	18 ± 15.3**c**	0 ± 0.0**F**	0 ± 0.0**F**	9 ± 2.3**EF**	25 ± 1.0**DE**
Q3G stearate	55 ± 6.1**bc**	74 ± 7.1**ab**	100 ± 16.9**a**	100 ± 3.6**a**	16 ± 2.5**E**	20 ± 5.2**DE**	69 ± 2.3**AB**	78 ± 3.1**A**
Q3G oleate	53 ± 8.5**bc**	56 ± 8.8**bc**	55 ± 4.1**bc**	63 ± 6.0**bc**	11 ± 3.4**EF**	30 ± 4.1**D**	43 ± 9.7**CD**	49 ± 14.4**C**
Q3G linoleate	47 ± 2.2**bc**	66 ± 0.6**b**	60 ± 8.1**bc**	73 ± 4.0**ab**	0 ± 0.0**F**	3 ± 1.0**F**	45 ± 2.0**CD**	67 ± 0.3**AB**
Q3G α-linolenate	13 ± 6.7**bc**	15 ± 0.1**c**	34 ± 4.0**c**	64 ± 3.0**c**	12 ± 1.4**EF**	24 ± 6.5**DE**	51 ± 1.3**BC**	63 ± 0.6**B**
Q3G eicosapentaenoate	42 ± 3.2**bc**	56 ± 5.4**bc**	63 ± 17.3**bc**	65 ± 12.2**bc**	0 ± 0.0**F**	0 ± 0.0**F**	13 ± 1.9**EF**	32 ± 1.8**D**
Q3G docosahexaenoate	49 ± 5.0**bc**	43 ± 4.0**bc**	54 ± 19.3**bc**	100 ± 8.4**a**	27 ± 9.5**DE**	26 ± 3.8**DE**	53 ± 0.8**BC**	58 ± 8.0**BC**

Primary oxidation was induced by adding AAPH and incubated at room temperature for 40 min and oxidation was determined by ferric thiocyanate test. Induction of secondary oxidation in bulk fish oil was achieved by incubating for 3 h at 50 °C in a shaking incubator and oxidation was determined by TBARS assay. Data from primary and secondary oxidation were statistically analyzed separately and presented as mean ± standard deviation. Different letters (a,b,c… and A,B,C…) denote significant differences among test compounds and concentrations, for % inhibition of primary and secondary lipid oxidation, respectively (*p* ≤ 0.05).

**Table 4:** Inhibition of human LDL oxidation *in vitro* in relation to the concentration of the test compounds.

Compound	% Inhibition (Relative to the Control)							
	Cu^2+^-Induction				AAPH Derived Peroxyl Radical–Induction			
	Concentration (µmol·L^−1^)				Concentration (µmol·L^−1^)			
	1	10	100	500	1	10	100	500
Q3G	7 ± 4.5**c**	20 ± 9.2**bc**	20 ± 1.8**bc**	33 ± 8.6**ab**	10 ± 2.6**C**	10 ± 2.7**C**	16 ± 7.2**C**	29 ± 2.0**C**
Q3G stearate	4 ± 9.4**c**	14 ± 7.5**bc**	21 ± 8.6**bc**	29 ± 4.2**b**	4 ± 5.9**C**	11 ± 5.0**C**	14 ± 7.5**C**	27 ± 8.5**C**
Q3G oleate	8 ± 7.4**c**	18 ± 5.8**bc**	14 ± 5.3**bc**	28 ± 2.8**bc**	12 ± 7.2**C**	9 ± 2.8**C**	15 ± 3.9**C**	17 ± 13.6**C**
Q3G linoleate	14 ± 3.4**bc**	27 ± 7.8**bc**	29 ± 5.9**b**	34 ± 6.3**ab**	5 ± 4.7**C**	15 ± 7.8**C**	23 ± 5.6**C**	25 ± 5.4**C**
Q3G α-linolenate	40 ± 5.6**ab**	34 ± 5.1**ab**	31 ± 2.9**ab**	31 ± 3.5**ab**	28 ± 9.5**AB**	23 ± 4.3**AB**	23 ± 2.0**AB**	34 ± 6.3**AB**
Q3G eicosapentaenoate	20 ± 8.8**bc**	43 ± 6.8**ab**	45 ± 3.1**ab**	51 ± 2.6**a**	21 ± 3.8**A**	36 ± 6.7**A**	38 ± 1.7**A**	41 ± 2.9**A**
Q3G docosahexaenoate	7 ± 5.5**c**	14 ± 7.9**bc**	27 ± 6.7**bc**	43 ± 1.4**ab**	13 ± 3.7**BC**	9 ± 8.1**BC**	30 ± 10.0**BC**	27 ± 8.1**BC**

Data from Cu^2+^- and peroxyl radical-induced LDL oxidation were statistically analyzed separately and presented as means ± standard deviation. Different letters (a,b,c… and A,B,C…) denote significant differences among test compounds and concentrations, for % inhibition of Cu^2+^- and peroxy radical- induced oxidation, respectively (*p* ≤ 0.05).

### 3.2. Inhibition of Oxidation in Human LDL

The ability of the compounds to inhibit the plasma LDL oxidation *in vitro* represents the antioxidant capacity of the compounds to deal with cellular oxidative stress. Over 40% inhibition for Cu^2+^-induced oxidation has been shown by the EPA derivative of Q3G at 10 µmol·L^−1^ and the DHA derivative at 500 µmol·L^−1^ (Table 4). For the AAPH derived peroxyl radical-induced oxidation, the selected concentration range did not demonstrate a dose dependent effect, but the EPA and ALA derivatives were more effective than Q3G in protecting the plasma LDL from oxidation. EPA derivative of Q3G, however, showed the highest inhibition in both experiments at concentrations of 500 µmol·L^−1^.

## 4. Discussion

Presently, there is a growing interest in plant polyphenols, especially flavonoids, which can be applied as natural antioxidants in foods, pharmaceuticals and cosmetics [21]. Their application can be further enhanced by modifying the flavonoid structure by esterification [7]. In this study, Q3G was successfully acylated with six different long chain fatty acids (C_18_ to C_22_) by lipase-catalyzed esterification. In all the reactions, the primary hydroxyl of the glycosyl group of the Q3G molecule was esterified with long chain fatty acids having various degrees of unsaturation (unsaturated 18:0, mono- 18:1 and poly-unsaturated 18:2; 18:3; 20:5; 22:6). The esterification of flavonoid compounds, using *Candida antarctica* B lipase as a very effective biocatalyst for various acyl donors, is widely reported in the literature. Naringin [22], chlorogenic acid [23], rutin [8], quercetin [14], Q3G [10], hesperidin [24] and esculin [13] are some of the extensively used flavonoids in esterification studies, with different acyl donors, such as palmitic acid, OLA, STA, lauric acid, myristic acid, γ-linolenic acid and hexadecanedioic acid.

However, the antioxidant ability of the flavonoids is highly dependent on their chemical structure; type, position and number of the functional groups, and they behave differently in different media [16]. In the literature, lipophilicity has demonstrated its effect on the antioxidant activity in different ways. Improved lipophilicity of an antioxidant molecule could result an enhanced, a similar or a decreased activity compared to that of parent compound. The alkyl esters of caffeic acid displayed enhanced antiradical activity, while dihydrocaffeic acid showed a decreased effect [25]. Lipophilisation of rutin molecule using fatty acids had no effect on radical scavenging capacity of rutin [26]. Further, it can be explained that accessibility of hydroxyl groups to the free radicals is obstructed by the steric hindrance created by long acyl chains of fatty acids and it decreases the radical scavenging ability of the synthesized phenolipids. Moreover, the considerably different reaction conditions used in the antioxidant assays make it difficult to draw unifying conclusions [27].

Oxidation stability is a parameter with greater attention and significance, in terms of evaluating the quality of lipid based foods, which are highly prone to oxidative deterioration. In the present study, the effects of enhanced lipophilicity of these synthesized phenolipids on inhibition of lipid oxidation were determined using two lipid based food model systems of PUFA-rich fish oil; bulk oil and oil-in-water emulsion. In oil-in-water emulsion systems, the phenolipids acted as more effective antioxidants than their parent Q3G. However, Q3G acted as more effective antioxidant for the inhibition of oxidation in bulk oil. The polar paradox theory explains the behavior of antioxidants in different media; the lipophilic antioxidants are more effective in inhibiting oxidation in less lipophilic media and hydrophilic antioxidants are more effective in inhibiting oxidation in more lipophilic media [28,29]. This is further explained using the interfacial phenomenon; lipophilic antioxidants locate towards the oil-water interface in oil-in-water emulsions, the location where the oxidation is more prevalent, and thereby protect the oil phase from oxidation. Hydrophilic antioxidants move into the aqueous phase and this dilution makes them not sufficiently effective for protecting the oil phase [30]. In bulk oil systems, oxidation is believed to take place in the association colloids, formed by surface active molecules in the presence of a small quantity of water in bulk oil [31].

There are a number of previous studies, which have exhibited the effect of the lipophilicity of antioxidants in bulk oil and emulsion model systems. It has been found, using a series of alkyl ester derivatives of tyrosol and hydroxytyrosol, that the lipophilic esters are weaker antioxidants in bulk oil [32]. Further it was reviewed in [30] that esterification with long hydrocarbon chains changed the partitioning behavior of chlorogenic acid and increased its ability to concentrate at oil-water interface. Therefore, it is apparent that esterification improves the lipophilicity of the antioxidants and the lipophilic and hydrophilic antioxidants have different effectiveness in different media. According to the explanations used in the polar paradox theory, it is understood that change in lipophilicity of the antioxidant molecules changes their physical location in different food matrices, affecting the effectiveness of the antioxidants. The observations in the present study indicate that esterification with long chain hydrocarbons improved the lipophilicity of the Q3G which caused the Q3G ester to be orientated with the lipophilic long chain fatty acid embedded in the oil and the polar Q3G in the aqueous phase surrounding the oil droplet. This means that the polar Q3G remains at the oil-water interface which yields protection from the oxidation of the droplet.

It has been explained that the polar paradox theory is applicable only in certain concentration ranges since the effect of solubility starts dominating over the interfacial phenomenon [30]. Therefore, in lipophilic media, more lipophilic antioxidants are effective at the lower concentrations and more hydrophilic antioxidants are effective at their higher concentrations. In the present study, Q3G was not effective in lower concentrations in the bulk oil and it can be due to solubility effect, which is more dominating at the lower concentrations. Since, these phenolipids are more lipophilic than Q3G, they are soluble in the lipid matrix playing a dominating role in inhibiting oxidation. Further, Q3G was more effective at its higher concentrations, at which the interfacial phenomenon dominates over the solubility effect. Moreover, long chain fatty acids including n-3 and n-6 PUFA have been used in this study and are known to have numerous health advantages. Therefore, in addition to the protection from oxidation, these novel molecules may provide clinical advantages.

The observations in the LDL oxidation model, reporting greater inhibition by several phenolipids compared to the Q3G, are supported by the previous study [6], which demonstrated that ethyl esterification of phenolic acids made them better antioxidants in inhibiting LDL oxidation. The previous studies have demonstrated the use of polyphenols including flavonoids as the inhibitors of LDL oxidation [30,33,34]. Moreover, the beverages prepared from flavonoids-rich fruits, such as red wine and grape juice, have been found to inhibit the human LDL oxidation *in vitro* [35,36,37]. Our present study demonstrated that increasing lipophilicity of the Q3G makes them more potent antioxidants in inhibiting the LDL oxidation. In addition, it has been postulated that an increase in lipophilicity of the ethyl esters facilitates the incorporation of the antioxidant molecules through the lipid layer of the LDL particles [6]. To better understand the mechanisms which make the incorporating of these esters more feasible, further studies are needed.

## 5. Conclusions

It can be concluded that all the derivatives of Q3G showed more than 30% inhibition in secondary oxidation and more than 50% inhibition in primary oxidation in the oil-in-water emulsion. The synthesized compounds were more effective in inhibiting both primary and secondary oxidation in oil-in-water emulsions than Q3G. However, Q3G reported 50%–60% inhibition at 10 mM concentration in bulk oil. These modified compounds were not significantly effective in bulk oil system, when compared with Q3G (*p* ≤ 0.05). Therefore, these synthesized phenolipids have a potential for use as effective antioxidants in PUFA containing emulsion based foods such as mayonnaise, salad dressings, and sour cream. The EPA and DHA derivatives of Q3G exhibited more than 50% inhibition in Cu^2+^-induced LDL oxidation. The EPA and ALA derivatives were more effective in AAPH derived peroxyl radical-induced oxidation than Q3G (*p* ≤ 0.05). Therefore, these novel phenolipids can be introduced as potential drug candidates for pathogenic conditions, such as atherosclerosis and related diseases. Further research is required to understand the biological functions of the modified Q3G.

## Abbreviations

STA: Stearic acid (18:0)
OLA: Oleic acid (18:1n-9)
LNA: Linoleic acid (18:2n-6)
ALA: Alpha-linolenic acid (18:3n-3)
EPA: Eicosapentaenoic acid (20:5n-3)
DHA: Docosahexaenoic acid (22:6n-3)
PUFA: Polyunsaturated fatty acid(s)
AAPH: 2,2'-Azobis(2-amidinopropane) dihydrochloride
BHT: Butylated hydroxytoluene
LDL: Low density lipoprotein
Q3G: Quercetin-3-*O*-glucoside
TBA: 2-Thiobarbituric acid
TBARS: Thiobarbituric acid reactive substances
TCA: Trichloroacetic acid
